# Lentinan and β-glucan extract from shiitake mushroom, *Lentinula edodes*, alleviate acute LPS-induced hematological changes in mice

**DOI:** 10.22038/IJBMS.2023.67669.14820

**Published:** 2023

**Authors:** Mojdeh Jafari, Mohammad Hossein Boskabaday, Seyed Abdolrahim Rezaee, Sharareh Rezaeian, Sepideh Behrouz, Rezvan Ramezannejad, Hamid Reza Pourianfar

**Affiliations:** 1 School of Medicine, Mashhad University of Medical Sciences, Inflammation and Inflammatory Diseases Research Centre, Mashhad, Iran; 2 Department of Physiology, Faculty of Medicine, Mashhad University of Medical Sciences, Mashhad, Iran; 3 Industrial Fungi Biotechnology Research Department, Research Institute for Industrial Biotechnology, Academic Center for Education, Culture and Research (ACECR)- Khorasan Razavi Branch, Mashhad, Iran

**Keywords:** β-glucans, Hematological parameters, Inflammation, Inhalation, Lentinan, Lipopolysaccharide, Shiitake

## Abstract

**Objective(s):**

Immunomodulatory activity of β-glucans of shiitake mushroom (*Lentinula edodes*) has been known. We investigated whether β-glucans from *L. edodes* would attenuate the acute effects of lipopolysaccharides (LPS) on peripheral hematological parameters in mice.

**Materials and Methods:**

An in-house β-glucans extract (BG) prepared from fruiting bodies of shiitake mushroom *L. edodes* was chemically measured and characterized using spectrophotometry and HPLC. Male BALB/c mice directly inhaled aerosolized LPS of 3 mg/ml and were treated with BG or commercial β-glucan (known as lentinan; LNT) (10 mg/kg bw) at 1 hr before or 6 hr after LPS inhalation. The blood samples were collected by cardiac puncture from euthanized mice at 16 hr post-treatment.

**Results:**

The results showed a significant reduction in levels of blood parameters, including red blood cells (RBC), hemoglobin (HGB), hematocrit (HCT), and platelets (PLT); and a significant increase in blood lymphocyte counts in LPS-treated mice as compared with the control mice (*P≤*0.05). Total white blood cells, neutrophils, and monocyte counts did not show any significant difference among the groups. Treatment of LPS-challenged mice with LNT or BG significantly increased the levels of RBC, HGB, HCT, and PLT; and reduced blood lymphocyte counts as compared with LPS-treated mice (*P≤*0.05).

**Conclusion:**

These findings suggest that β-glucans from *L. edodes* might be effective in attenuating the effects of inhaled LPS on peripheral blood parameters. Thus, these findings might be useful in acute inflammatory diseases particularly pulmonary infectious diseases in which the hematological parameters would be affected.

## Introduction

Lipopolysaccharide (LPS) also known as endotoxin is the most abundant major component within the gram-negative bacteria cell walls which is involved in the activation of the CD14/TLR4 receptor on the monocytes and macrophages and regulation of the inflammatory cytokines (1). Such inductive effect of LPS has led to the use of this compound in animal models of diseases such as sepsis, mastitis, enteritis, acute lung injury (ALI), and various cancer types (2-6). Administration of LPS in rodent animals is usually carried out using two main routes, including injection (intravenous or intraperitoneal) and pulmonary delivery of LPS (3, 7). The latter can be modeled through either tracheal instillation or inhalation where the alveolar epithelium is the primary damaged structure (8). Pulmonary delivery of LPS may lead to ALI (9), causing infiltration of excessive neutrophils into the lung tissues followed by the release of pro-inflammatory cytokines of endothelial and epithelial lung injury (10). LPS-simulated ALI also increased the content of white blood cells in the lung (11). However, very little is known regarding the effects of pulmonary delivery of LPS on hematological parameters of blood in a murine model. 

β-glucans are abundant in the cell wall of mushrooms and consist of long or short-chain polymers of glucose subunits with β-1,3 and β-1,6 linkages that are responsible for the linear and branching structures, respectively (12). The most studied health-related effects of mushroom β-glucans include their ability to involve in modulating the immune system (12). Lentinan, a β-glucan with a 1,3 linkage derived from shiitake mushroom (*Lentinula edodes*) has been widely studied mainly for its immunomodulatory activity. Lentinan triggers signaling pathways, such as MAPK, NF-κB, and Syk-PKC, via binding pattern recognition receptors (TLRs, Dectin-1) and the complement receptor type 3 (CR3, also known as CD11b/CD18) on the membrane of various immune cells particularly natural killers, macrophages, and T cells (13-15). Such effects have also been demonstrated for polysaccharide fractions and extracts containing β-glucans isolated from shiitake mushrooms (14, 16, 17).

A number of previous studies have confirmed that lentinan or β-glucans extracts from shiitake mushroom can attenuate *in vitro* LPS-induced pro-inflammatory cytokines and oxidative stress (17), and improves *in vivo* LPS-induced mastitis (6), tumor growth (18), and lethality in mice (19). However, no animal study has been undertaken to investigate the alleviative effects of lentinan or β-glucans extracts obtained from shiitake mushrooms in response to LPS delivered through the pulmonary route. Also, very little is known about how lentinan or β-glucans extracts neutralize or ameliorate LPS-induced damages on blood parameters in a murine model. Consequently, the present study aimed to evaluate whether or not a commercial lentinan and an in-house β-glucans extract from shiitake mushroom would attenuate the negative effects of LPS inhaled into the lung on hematological parameters in mice.

## Materials and Methods

***Reagents***

LPS (*Escherichia coli*, L2630) and Lentinan (S5083) were purchased from Sigma-Aldrich and Selleckchem (USA), respectively. Mushroom and Yeast Beta-glucan kits (K-YBGL 11/19) were purchased from Megazyme (Ireland). 

***Production of in-house polysaccharide extract***

Fruiting bodies of shiitake mushroom, *L. edodes* (strain no. M3102, the Mycelia company, Deinze, Belgium) were prepared in the mushroom laboratory of Industrial Fungi Biotechnology Research Department, Academic Center for Education, Culture, and Research (ACECR)-Mashhad, Iran. Mature fruiting bodies were harvested and immediately stored in a -80 ^°^C freezer for 48 hr before being subjected to quick-freezing with liquid nitrogen according to a protocol described previously (20). The frozen samples were transferred to a freeze drier (Ferdowsi University’s central laboratory, Mashhad, Iran) under 0.1 mbar for 72 hr until a fine powder was obtained. The lyophilized samples were stored at -20 ^°^C until use. Polysaccharide extract (PE) of the lyophilized shiitake mushroom was produced based on a hot water method (21, 22). Distilled water with a ratio of 1 to 10 was added to 100 g of the powder and autoclaved for 30 min at 120 ^°^C under the pressure of 1 bar. After cooling, the solution was centrifuged at 10,000 rpm for 10 min. The supernatant was mixed with 96% ethanol and placed at 4 ^°^C for 24 hr. The extract was centrifuged at 10,000 rpm for 10 min. The resultant precipitate considered PE was subjected to freeze-drying as described above.

***Chemical analysis of*** ***β*** ***-*** ***glucan***

Quantification of the β-glucan present in the lyophilized PE was performed at 510 nm using a spectrophotometer based on the manufacturer’s instructions of a special mushroom and yeast β-glucan assay kit (cat. No. K-YBGL, Megazyme International, Wicklow, Ireland). In addition, High-performance liquid chromatography (HPLC) analysis of β-glucan was performed using a single four-solvent pump HPLC Model 1100 of Agilent (USA) with a RID detector (Pars Knowledge Pars Company, Isfahan, Iran). The C18 column had a particle size of 4.6 µm in 250 mm and a half-centimeter ODS column. The mobile phase of 100% deionized water with a flow rate of 1 ml/min was isocratically applied with a refractive index detector at ambient temperature. Commercial lentinan was also subjected to HPLC analysis using the same method as described for PE containing β-glucan. The amount of β-glucan in the PE was calculated using the line equation obtained from drawing the standard curve of lentinan (y=156608x-7150.7).

***Animals***

Male Balb/C mice (aged 7 weeks, weighing 20 g on average) were provided by the animal housing laboratory of Mashhad University of Medical Sciences (Mashhad, Iran). The experimental protocol for all mice was approved by the Animal Ethics Committee of the Iranian Academic Center for Education, Culture, and Research, Mashhad (code no. IR.ACECR.JDM.REC.1399.010). The animals were kept in a controlled environment with free access to food and water. All mice used in this research were treated humanely according to institutional guidelines for animal welfare, with due consideration to the alleviation of distress and discomfort.

***LPS preparation and aerosol exposures***

LPS was freshly reconstituted in 3 mg/ml of phosphate-buffered saline (PBS). The LPS solution was aerosolized with an air nebulizer in the animal housing laboratory of Mashhad University of Medical Sciences (Mashhad, Iran) according to a previous protocol with mice (23), with modifications following a pilot study test. Every five mice were placed in a cylindrical chamber with all output connected to the air nebulizer containing 1.5 ml of the LPS solution. Mice inhaled the aerosolized LPS for 20 min and remained inside the chamber for another 20 min while the air nebulizer was turned off. Control mice were exposed to 1.5 ml of aerosolized PBS under the same condition as described for LPS-treated mice.

***Preparation of injectable treatment***

Lentinan or the PE containing β-glucan (20 mg) was reconstituted in 500 μl of 20% (v/v) DMSO in PBS to make a stock of 40 mg/ml. The stock solution was diluted with PBS to reach a concentration of 2 mg/ml, while the concentration of DMSO became equal to 1% v/v. The working solution was filtered through a 0.22-micron filter and placed at 4 °C until use. Then, 100 µl of the cold working solution was used for intraperitoneal injection in each 20-g mouse. This amount of injectable lentinan or the PE containing β-glucan was equal to 10 mg/kg of mouse bw which has been shown to be safe and did not have any negative effect on the survival rate of mice in a previous study (19).

***Study design***

Four study groups (n=5 each) were designed as follows:

• Control: Mice exposed to aerosolized PBS and euthanized after 16 hr.

• Sham: Mice exposed to aerosolized LPS (dose: 3 mg/ml in PBS; 1.5 ml final volume) and euthanized after 16 hr. These mice were treated with PBS alone. 

•^ LPS^LNT: Mice treated with lentinan (10 mg/kg bw) at 6 hr after (treatment) or 1 hr before (prophylaxis) exposure to aerosolized LPS (dose: 3 mg/ml in PBS; 1.5 ml final volume). Mice were euthanized 16 hr after exposure to LPS. 

•^ LPS^BG: Mice treated with the PE containing β-glucan (10 mg/kg bw) at 6 hr after (treatment) or 1 hr before (prophylaxis) exposure to aerosolized LPS (dose: 3 mg/ml in PBS; 1.5 ml final volume). Mice were euthanized 16 hr after exposure to LPS.

***Blood collection***

Mice were deeply anesthetized by 100 µl of a mixture of 10% xylazine and 10% ketamine at the ratio of 3:7. The blood samples were obtained by cardiac puncture. In brief, the anterior abdominal wall was split and then the diaphragm muscle was removed. The beating heart was gently accessed and cardiac blood was extracted by a 22G needle from the right and left ventricles. About 500 µl of the blood was poured into EDTA-treated tubes and kept shaking to avoid agglutination until they were subjected to cytometry. A complete blood count (CBC) of the blood samples was done by a Hematology Analyzer (Siemens, ADVIA^®^ 2120i) in the medical central laboratory of the Iranian Academic Center for Education, Culture, and Research (ACECR), Mashhad, Iran. Table 1 shows the hematological parameters measured by the Hematology Analyzer.

***Statistical analysis***

Data were subjected to one-way ANOVA using SPSS software version 25. Mean values were compared by Duncan’s Multiple Range Test and reported as means±standard deviation. A probability of *P*≤0.05 was considered to be significant. Graph prism version 9 was utilized to draw graphs. 

## Results

***Confirmation of*** ***β*** ***-*** ***glucans*** ***in the in-house extract ***

**Figure 1:**
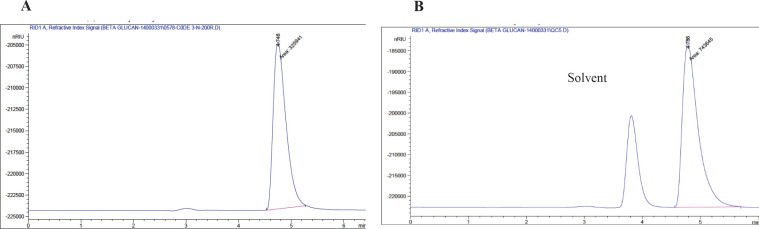
HPLC chromatogram of commercially available nearly pure lentinan and a polysaccharide extract containing β-glucan isolated from fruiting bodies of shiitake mushroom

The extraction efficiency of PE was found to be 9.12% of the shiitake mushroom powder (dw). The mushroom and yeast β-glucan assay kit-based quantification of glucans showed that PE contained 27% β-glucan and 5% α-glucan. Further HPLC analysis revealed that PE produced a clear and strong peak with a retention time (RT) of 4.748 min (Figure 1a). A similar peak with an RT of 4.786 was also obtained with LNT (Figure 1b) as a nearly pure β-glucan extracted from shiitake mushroom. According to the peak area quantification, PE contained 27.5% β-glucan** (**dw) which confirmed the kit-based findings. 

***RBC, hematocrit, and MCV contents***

**Figure 2:**
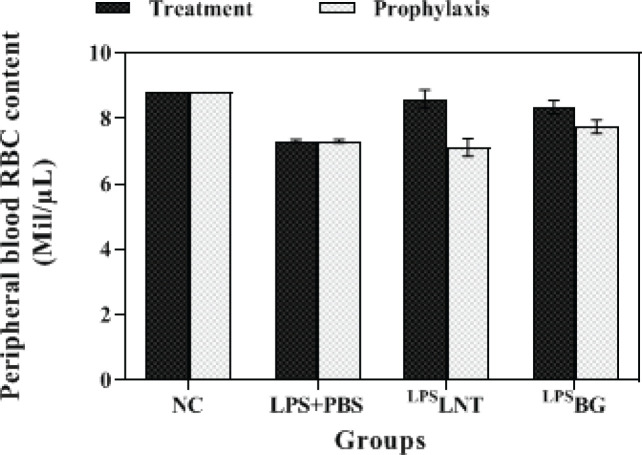
Effects of β-glucans from shiitake mushroom on improving red blood cell (RBC) levels in peripheral blood of mice challenged with LPS

As depicted in Figure 2, inhalation of LPS by healthy mice significantly reduced their RBC amount compared with the control group (*P*≤0.05). LPS-exposed mice treated with LNT or BG displayed a significantly higher level of RBC compared with the sham group (*P*≤0.05). In addition, there was no statistically significant difference in RBC levels between the control and LPS-exposed mice treated with LNT, with RBC levels in each of these groups being 8.8 and 8.6 million/μl, respectively (*P*≥0.05). However, the RBC content in LPS-exposed mice treated with BG was significantly lower than in the control mice (*P*≤0.05). 

The prophylactic groups of mice exhibited different results as compared with the treatment groups. Pre-treatment of mice with LNT or BG did not compensate for the decrease in RBC count caused by LPS inhalation compared with the control mice (Figure 2). 

**Figure 3:**
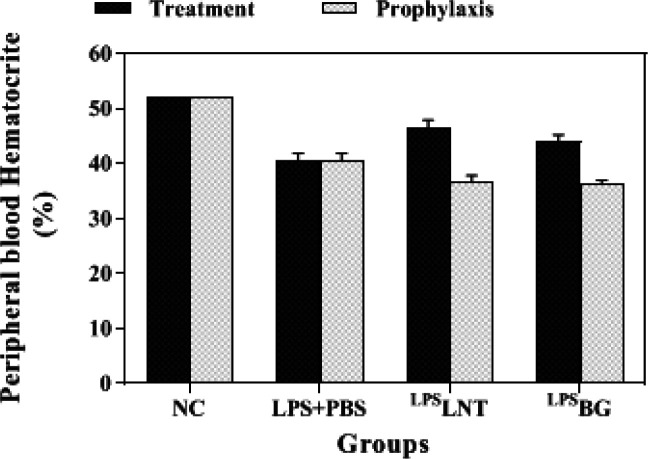
Effects of β-glucans from shiitake mushroom on changes in hematocrit percentages in peripheral blood of mice challenged with LPS

As expected from the results of the RBC count, inhalation of LPS by healthy mice significantly reduced their hematocrit levels compared with the control group (*P*≤0.05) (Figure 3). The hematocrit content of 40.6% in the LPS-exposed mice treated with PBS was significantly lower than that of LPS-exposed mice treated with LNT (*P*≤0.05). However, the hematocrit content of neither LNT nor BG groups reached that of the control group (*P*≤0.05). In the prophylaxis tests, no alleviative effect of LNT or BG was observed in the LPS-challenged mice as compared with the control group (Figure 3). 

**Figure 4:**
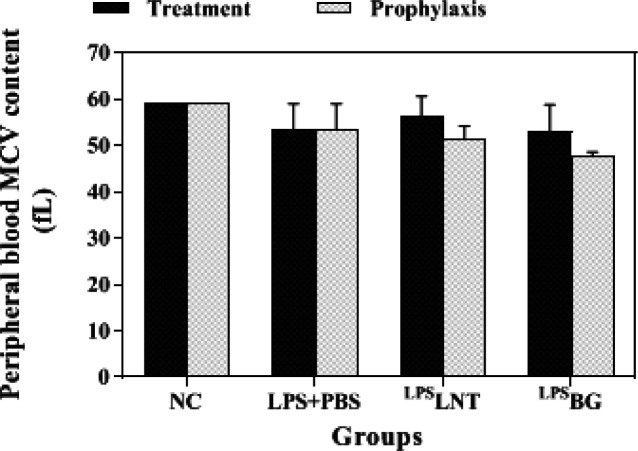
Effects of β-glucans from shiitake mushroom on improving mean corpuscular volume (MCV) levels in peripheral blood of mice challenged with LPS

The average size of RBC indicated as mean corpuscular volume (MCV) decreased in LPS-exposed mice, but this reduction was not significant (*P*≥0.05). Although treatment of LPS-exposed mice with LNT or BG apparently increased MCV, the difference was not statistically significant at *P*-value less than 0.05 (Figure 4). Meanwhile, pre-treatment of mice with BG in the prophylactic groups caused a reduction in MCV as compared with the sham group (Figure 4).

***Hemoglobin and MCHC contents***

**Figure 5:**
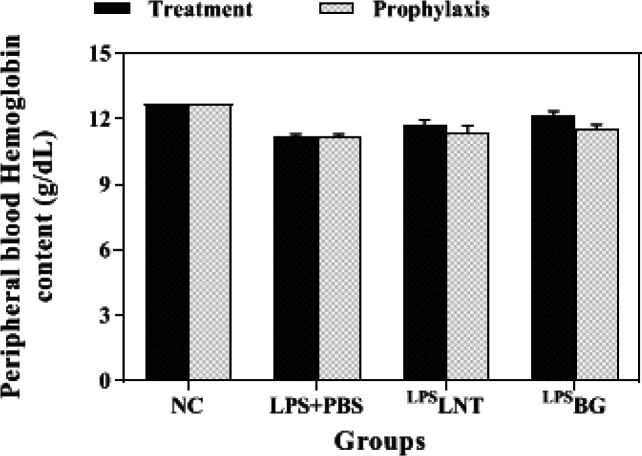
Effects of β-glucans from shiitake mushroom on improving hemoglobin levels in peripheral blood of mice challenged with LPS

Hemoglobin (HGB) levels of healthy mice exposed to LPS inhalation were notably lower than the control group (*P*≤0.05). Although treatment of LPS-exposed mice with LNT increased their HGB levels, only BG significantly increased the HGB content of LPS-challenged mice from 11.18 to 12.14 g/dL (*P*≤0.05). However, neither LNT nor BG reaches the count of hemoglobin in the control group statistically (*P*≤0.05). Similar to the RBC count, Pre-treatment of mice with LNT or BG did not significantly alleviate the reducing effect of LPS inhalation on mice hemoglobin levels (Figure 5).

**Figure 6:**
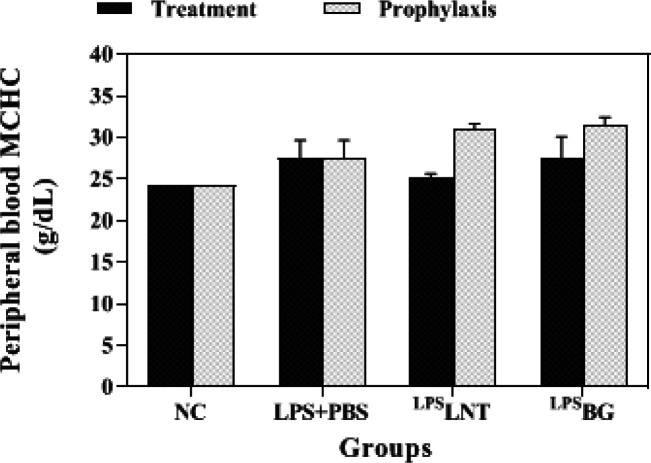
Effects of β-glucans from shiitake mushroom on improving mean corpuscular hemoglobin concentration (MCHC) levels in peripheral blood of mice challenged with LPS

The mean corpuscular hemoglobin concentration (MCHC) measures the average concentration of HGB in the RBCs. The findings showed a significant increase in MCHC following LPS inhalation in mice. Treatment with LNT was able to significantly reduce this index to the control group level. In the prophylaxis group, no significant difference in the level of MCHC was observed between the sham and the treatment groups (Figure 6). 

***Platelet and MPV contents***

**Figure 7:**
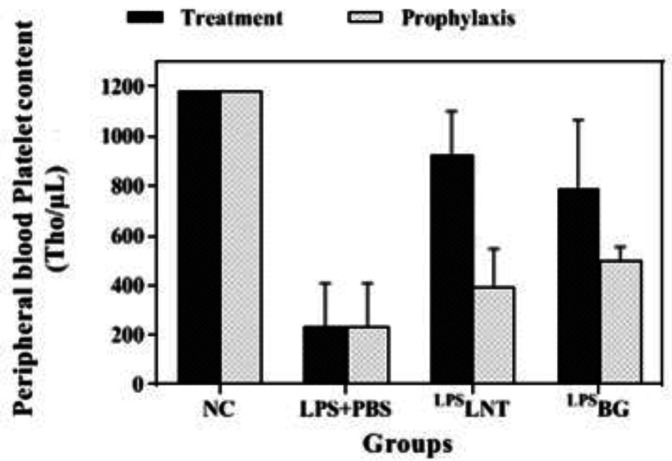
Effects of β-glucans from shiitake mushrooms on improving platelet contents in peripheral blood of mice challenged with LPS

**Figure 8:**
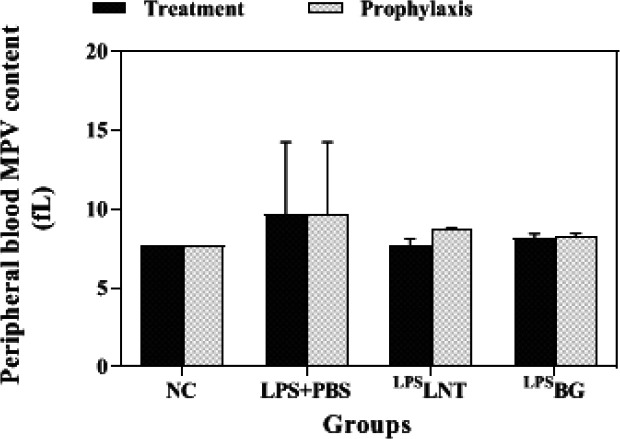
Effects of β-glucans from shiitake mushrooms on improving mean platelet volume (MPV) levels in peripheral blood of mice challenged with LPS

Inhalation of LPS by healthy mice considerably reduced their platelet levels compared with the control group (*P*≤0.05). Post-treatment or pre-treatment of LPS-exposed mice with LNT or BG significantly increased the platelet levels as compared with the sham group (*P*≤0.05). However, no treatment could increase this index as much as that of the control group (Figure 7). Despite changes in the number of platelets, there was no statistically significant change in the average size of platelets indicated as mean platelet volume (MPV) among the groups (Figure 8).

***Cardiac WBC and differential count***

Healthy mice receiving LPS by inhalation showed a significant increase in their cardiac lymphocyte counts compared with the control group (*P*≤0.05) (Table 2). In contrast, lymphocytes reduced to the levels observed in the control group after treatment of LPS-exposed mice with LNT or BG (Table 2). Cardiac total WBC, neutrophils, and monocytes did not show any significant changes between the treatment groups. Pre-treatment of mice with LNT or BG decreased the counts of neutrophils and lymphocytes as compared with the sham group. However, no change in monocyte levels was observed in the prophylaxis groups (Table 2). 

## Discussion

In this study, we used a murine model with direct inhalation of aerosolized LPS in order to assess its acute effects on peripheral blood parameters. Then, LPS-challenged mice were treated with a β-glucan extract directly obtained from fruiting bodies of shiitake mushrooms and a nearly purified commercially available β-glucan (known as Lentinan) derived from the same mushroom species. Overall, our findings for the first time revealed that pulmonary delivery of aerosolized LPS apparently decreased the levels of RBC, HGB, and HCT in the peripheral blood of mice. These results are in agreement with the findings obtained from intraperitoneal injection of LPS where a significant decline was observed in several peripheral blood parameters including RBC, HGB, HCT, MCHC, and PLT in mice (24). Recently it was also shown that injected LPS decreased cardiac RBC content in mice (5). In addition, RBC aggregation and deformability were also observed in 24 hr after IP injection of LPS in mice (25). Similar findings have also been obtained with rats where IP injection of LPS declined peripheral HGB (26, 27). 

This study also showed a decline in peripheral blood PLTs 16 hr after inhalation of LPS in mice. These findings are in perfect match with previous studies, where a rapid reduction in the number of circulating platelets occurred at 3 hr to 24 hr post-LPS injection in the peripheral blood of mice and rats (24, 26, 28-34). PLTs are non-nucleate blood cells with reported roles in hemostasis and immune responses by interacting with other cells of the immune system and by secreting inflammatory mediators (35). Therefore, PLT has been known as an important agent in inflammatory reactions, particularly in acute form. Since TLR4 is also expressed on the surface of PLTs, they possess a functional receptor for bacterial LPS-inducing inflammation (34). It has also been demonstrated that the effect of LPS on blood PLT levels depends on the time elapsed after LPS injection, so PLT depletion at early hours post-LPS injection has been shown to prevent LPS-induced rapid shock but also increase delayed lethality (34). Consistently with animal-based findings, increased mortality of septic patients has clinically been associated to reduce numbers of circulating PLTs (36). Taken together, the findings of the present study may suggest that direct inhalation of aerosolized LPS would have acute effects on peripheral blood parameters in mice in the same way as intraperitoneal or intravenous injection of LPS. 

This study also showed a considerable increase in total peripheral blood lymphocyte counts and no significant change in the levels of total WBC, neutrophils, and monocytes in response to pulmonary delivery of LPS in mice. These findings may contradict a previous work in which total WBC levels obtained from the blood of mice showed a marked increase at 4 hr after injection of LPS followed by a decrease during 12 hr (37). Contrarily, another study reported that the WBC count declined significantly at 3 hr after LPS injection and increased with time showing a partial normalization at 12 hr in rats and mice (31). Thus, the changes in the levels of total WBC and its differential cells in response to LPS observed in this study might be related to a specific inflammation pattern of these cells 16 hr after LPS inhalation, which could be different from the outcome of LPS injection. However, the result obtained from some studies showed that LPS is T cell-independent B cell mitogen and polyclonal activator in mice. Mitogens are substances that cause DNA synthesis, blast transformation, and ultimately the division of lymphocytes (38).

According to the current study, mice given shiitake-derived β-glucans (BG or LNT) after being exposed to aerosolized LPS had significantly higher levels of RBC, HGB, and PLT and lower levels of lymphocytes in their peripheral blood. These findings might be confirmed by previous observations that bacterial BG significantly increased the WBC count, RBC count, HCT, HGB, and PLT in males fed BG than in control rats (39). LNT is an immune activator that activates macrophages and lymphocytes, increases the chemotaxis of macrophages and the toxic response of lymphocytes to Yac-1 cells and P-815 cells, and antagonizes the carcinogenicity of BBN in mice (40). To the best of our knowledge, this is the first *in vivo* study to report the relieving effects of mushroom-derived β-glucans in relation to the hematological effects of LPS, although its immunomodulatory effects have been well understood *in vitro* (14, 16, 17) and *in vivo* (4, 6, 18, 19, 41, 42).

**Table 1:** Measured hematological parameters in peripheral blood of mice challenged with LPS in this study

Hematological parameters			
No.	Complete name	Abbreviation	Unit
1	Red Blood Cell	RBC	Mil/μl
2	Mean Corpuscular Volume	MCV	fL
3	Hematocrit	HCT	%
4	Hemoglobin	HGB	g/dl
5	Mean Corpuscular Hemoglobin Concentration	MCHC	g/dl
6	Platelet	PLT	Tho/μl
7	Mean Platelet Volume	MPV	fL
8	White Blood Cell	WBC	count
9	Lymphocyte	-	count
10	Monocyte	-	count
11	Neutrophil	-	count

Values are expressed as means±standard deviation generated from five mice in each group

LNT: Lentinan; BG: Polysaccharide extract containing β-glucans

Values are expressed as means±standard deviation generated from five mice in each group

LNT: Lentinan; BG: polysaccharide extract containing β-glucans

Values are expressed as means±standard deviation generated from five mice in each group

LNT: Lentinan; BG: Polysaccharide extract containing β-glucans

Values are expressed as means±standard deviation generated from five mice in each group

LNT: Lentinan; BG: Polysaccharide extract containing β-glucans

Values are expressed as means±standard deviation generated from five mice in each group

LNT: Lentinan; BG: Polysaccharide extract containing β-glucans

Values are expressed as means±standard deviation generated from five mice in each group

LNT: Lentinan; BG: Polysaccharide extract containing β-glucans

Values are expressed as means±standard deviation generated from five mice in each group

LNT: Lentinan; BG: Polysaccharide extract containing β-glucans

**Table 2:** Effects of β-glucans from shiitake mushroom on improving total WBC and differential count in peripheral blood of mice challenged with LPS (n=5 each)

	**Control**	**LPS+PBS**	^LPS^ **LNT**	^LPS^ **BG**		**Control**	**LPS+PBS**	^LPS^ **LNT**	^LPS^ **BG**
**Total WBC**	2200^a^	2683^a^	1806^a^	2220^a^		2200^a^	2683^a^	2000^a^	1640^a^
**Neutrophil**	130^a^	188^a^	120^a^	158^a^		130^b^	188^a^	88.33^b^	58.00^b^
**Lymphocyte**	1082^b^	2042^a^	834^b^	1145^b^		1082^b^	2042^a^	630^b^	535^b^
**Monocyte**	704^a^	604^a^	492^a^	571^a^		704^a^	604^a^	735^a^	703^a^

Values shown are the means of five mice±standard deviation of the mean. In each row, the statistical lower-case letters including “a, b” indicate significant differences between the means at the statistical level of 5% or less (*P≤*0.05). Values followed by the same lower-case letters are not significantly different

LNT: Lentinan; BG: Polysaccharide extract containing β-glucans

## Conclusion

The evidence from this study for the first time demonstrates that β-glucans from shitake mushroom, *L. edodes,* may be effective in attenuating the effects of inhaled aerosolized LPS on blood parameters. These findings might be used for further research mimicking characteristics of diseases caused by aerosol or air-borne transmission of infectious microorganisms in which hematological parameters would be affected.

## Authors’ Contributions

MJ performed *in vivo* experiments, MHB discussed the results and strategy, SA RR discussed the results and strategy, SH R performed *in vitro* experiments, SB performed *in vivo* experiments, RR performed *in vivo* experiments and collected data, HP supervised and managed the study, performed *in vivo* experiments, and wrote the manuscript.

## Conflicts of Interest

The authors declare that they have no conflicts of interest.
